# The CALLY index for risk stratification of advanced cardiovascular-kidney-metabolic syndrome and mortality: a dual-cohort study from NHANES and a real-world clinical setting

**DOI:** 10.3389/fmed.2026.1811091

**Published:** 2026-06-26

**Authors:** Peiting Chen, Zhenjia Deng, Wei Zhou, Fangqi Zhu, Yanni Tan, Yuting Tang, Ruier Huang, Jian Wang, Xi Li

**Affiliations:** 1 Key Laboratory of Clinical Laboratory Medicine of Guangxi Department of Education, Department of Clinical Laboratory, the First Affiliated Hospital of Guangxi Medical University, Nanning, Guangxi, China; 2 Department of Laboratory Medicine, Wuming Hospital of Guangxi Medical University, Nanning, Guangxi, China

**Keywords:** all-cause mortality, CALLY index, cardiovascular-kidney-metabolic syndrome, dual cohort study, risk stratification

## Abstract

**Background:**

Cardiovascular Kidney Metabolic (CKM) syndrome is a complex systemic condition, but identifying patients at advanced stages (Stages 3 or 4) remains a clinical challenge due to the lack of readily available markers. The C-reactive Protein-Albumin-Lymphocyte (CALLY) index is a composite measure reflecting the axis of inflammation, nutrition, and immunity. However, its role across integrated advanced CKM risk stratification and all-cause mortality assessment, particularly with cross-population validation, remains insufficiently clarified.

**Methods:**

This study used a dual-cohort design. We analyzed a nationally representative population from the United States (NHANES, *N* = 11,690) and an independent clinical cohort from a Chinese hospital (*N* = 1,056) for external validation. The associations between the CALLY index and advanced CKM or all-cause mortality were assessed using survey-weighted logistic regression and Cox proportional hazards models. Non-linear dose-response relationships were evaluated using restricted cubic splines (RCS). We further measured the incremental value of the CALLY index over standard models through the Net Reclassification Improvement (NRI), Integrated Discrimination Improvement (IDI), and Decision Curve Analysis (DCA).

**Results:**

In the NHANES cohort, a higher CALLY index was associated with a lower risk of advanced CKM (Q4 vs. Q1: OR 0.70, 95% CI 0.54–0.90) and all-cause mortality (Q4 vs. Q1: HR 0.64, 95% CI 0.55–0.76). In the external validation cohort, participants in the highest CALLY quartile had lower odds of advanced CKM (Q4 vs. Q1: OR 0.41, 95% CI 0.28–0.62). Both cohorts showed a clear L-shaped non-linear association. Adding the CALLY index to conventional models significantly improved risk reclassification (NRI 0.1968 in NHANES and 0.4134 in the validation cohort, both *P* < 0.001). DCA also indicated that the CALLY-integrated model provided greater model-based net benefit across a range of threshold probabilities, despite relatively small changes in the area under the curve (AUC).

**Conclusion:**

The CALLY index is a simple, routinely available composite indicator that may support advanced CKM risk stratification and mortality risk assessment. It provided incremental risk-stratification information in this study and may help identify individuals at higher risk across the CKM spectrum.

## 1 Introduction

Cardiovascular Disease (CVD), Diabetes Mellitus (DM), and Chronic Kidney Disease (CKD) share closely interrelated pathophysiology (1, 2), underpinned by systemic low-grade inflammation, oxidative stress, and endothelial dysfunction (3, 4). Recognizing this, the American Heart Association (AHA) formally introduced the systemic concept of “CKM syndrome” in 2023 (5, 6). Although the prevention and management of CKM have garnered global attention, there is a distinct lack of convenient biomarkers that integrate these multidimensional pathological factors. Ideally, such biomarkers would be easily implementable in primary care settings to facilitate the early identification and risk stratification of advanced-stage CKM (Stages 3–4).

The CALLY index, derived from C-reactive protein (7, 8), serum albumin (9, 10), and peripheral lymphocyte count (11), simultaneously captures three critical dimensions: inflammatory burden, hepatic nutritional synthetic capacity, and adaptive immune surveillance. This index is characterized by its low cost and potential for widespread adoption (12). Initially developed for assessing cancer prognosis (13–17), recent studies have confirmed its predictive value for specific cardiovascular or metabolic outcomes (18, 19). Recent NHANES-based studies have also separately reported inverse associations of the CALLY index with advanced CKM syndrome and with mortality among individuals with CKM stages 0–3; however, these studies addressed adjacent but separate questions and remained confined to single-source NHANES evidence (20, 21). Whether it effectively reflects pathological evolution within the integrated CKM framework, particularly its relationship with advanced CKM and all-cause mortality, still lacks large-scale population-based evidence and real-world validation.

To address this gap, this study aims to comprehensively evaluate the clinical value of the CALLY index in the risk stratification of CKM. A dual-cohort design was employed: first, the independent association and potential nonlinear characteristics between the CALLY index and the risks of advanced CKM syndrome and all-cause mortality were systematically analyzed using nationally representative NHANES data. Subsequently, an independent Chinese hospital-based clinical cohort was introduced for external validation to examine the robustness and cross-population generalizability of this indicator in real-world scenarios. This cohort was intentionally selected because it differs substantially from NHANES in ethnicity, clinical setting, case mix, and EHR-based data structure, thereby allowing us to evaluate whether the observed association is transportable beyond a single-source community-based dataset. Unlike prior studies that mainly focused on isolated cardiovascular or metabolic outcomes, the study objectives are to determine whether the CALLY index can serve as a simple tool reflecting the homeostasis of the “inflammation–nutrition–immunity” axis, thereby aiding risk stratification across the CKM continuum and early identification of high-risk populations, and to provide an evidence base for individualized clinical follow-up and resource allocation at the population level. In addition, this study evaluates the incremental clinical usefulness of the CALLY index for advanced CKM risk stratification using AUC, NRI/IDI, and DCA, and externally examines the transportability of the observed association in an independent Chinese hospital-based cohort.

## 2 Materials and methods

### 2.1 Study population and design

**FIGURE 1:**
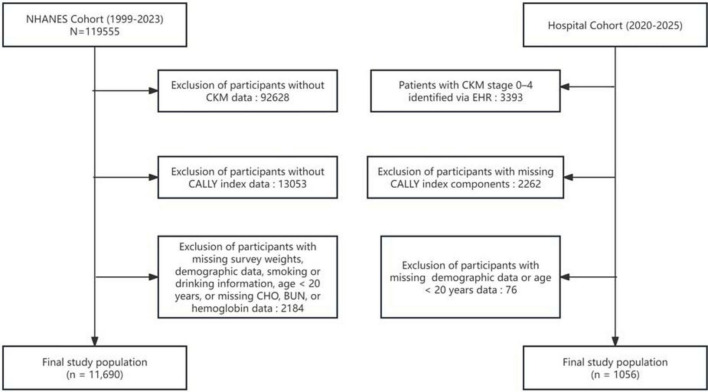
Flow chart of participant selection. NHANES, National Health and Nutrition Examination Survey; CKM, cardiovascular-kidney-metabolic; EHR, electronic health record; CHO, cholesterol; BUN, blood urea nitrogen.

This study employed a dual-cohort design, incorporating a primary analysis cohort from the NHANES and an external validation cohort from a tertiary hospital in China (Figure 1). The primary analysis was based on continuous cross-sectional data from NHANES. Initially, a total of 119,555 participants from the 1999–2023 cycles were screened. Participants were sequentially excluded if they had missing data for CKM staging variables, missing components of the CALLY index, or missing sampling weights and key covariates (e.g., smoking, alcohol consumption). Thus, the substantial reduction in sample size mainly reflected the strict data requirements for CKM staging, CALLY index calculation, survey design variables, and covariate adjustment. Following these rigorous exclusion criteria, the final analytical sample comprised 11,690 participants derived from 1999 to 2010 cycles. In this cohort, advanced CKM (Stages 3–4) was analyzed as a cross-sectional outcome, while all-cause mortality served as a longitudinal outcome, ascertained via linkage to the National Death Index records through December 31, 2019. The external validation cohort was retrospectively recruited from Wuming Hospital of Guangxi Medical University, China. Between 2020 and 2025, 3,393 adults (aged ≥ 20 years) were initially identified. To ensure data quality and comparability, patients with incomplete clinical data for CALLY index calculation, missing basic demographics, or evidence of severe acute infection (e.g., CRP > 10 mg/dL) indicative of non-chronic inflammatory states were excluded. The final external validation cohort consisted of 1,056 patients. For the hospital cohort, all logistic regression analyses were conducted using the same complete-case sample of participants with available CALLY index, advanced CKM status, age, sex, and BMI (*n* = 872). Given the absence of longitudinal follow-up, we restricted the analysis of this cohort to a cross-sectional validation of the relationship between the CALLY index and advanced CKM syndrome. We used this hospital-based cohort because it provides a complementary real-world clinical setting, with a distinct ethnic composition, disease spectrum, and data structure compared with NHANES, enabling an assessment of the external transportability of the observed advanced CKM association.

### 2.2 Definition of CKM syndrome

CKM syndrome staging (Stages 0–4) was strictly defined according to the 2023 American Heart Association (AHA) scientific statement (6). The staging continuum ranges from ideal health (Stage 0) to clinical CVD (Stage 4). Specifically, Stage 3 denotes high risk for CVD (e.g., equivalent risk variants of CKD or subclinical heart failure), while Stage 4 indicates established clinical CVD. This study focused on advanced CKM syndrome, defined as the composite of Stages 3 and 4, representing a population at significantly elevated risk for adverse cardiovascular events and mortality (6). In the NHANES cohort, race-specific BMI thresholds ( ≥ 25 kg/m^2^ for non-Asians; ≥ 23 kg/m^2^ for Asians) were applied to define excess adiposity. To align the hospital cohort with NHANES standards, we implemented a hierarchical prioritization algorithm based on the AHA 2023 criteria (6, 22), employing standardized proxy variables for missing EHR items (e.g., waist circumference). In the hospital cohort, CKM staging was implemented using a predefined top-down hierarchical algorithm (Stage 4 → Stage 0). When exact AHA-specified variables were unavailable in the electronic health record, clinically aligned proxy measures were used based on the closest available definitions. Cardiovascular and kidney disease were primarily ascertained using ICD-10 concept sets, and metabolic syndrome was operationalized using available components because waist circumference was not routinely recorded. Detailed staging algorithms and code repositories are available in Supplementary material and Supplementary Figure 1.

### 2.3 Definition of CALLY index and covariates

The CALLY index, a composite indicator of inflammation, nutrition, and immunity, served as the primary exposure variable. It was calculated using the formula:


$$T\,h\,e\,C\,A\,L\,L\,Y\,i\,n\,d\,e\,x=\left(\frac{A\,l\,b\,u\,m\,i\,n\,\left(g/L\right)\times L\,y\,m\,p\,h\,o\,c\,y\,t\,e\,C\,o\,u\,n\,t\,\left(10^{9}/L\right)}{C\,R\,P\,\left(m\,g/L\right)\times 10}\right)$$

Covariates were selected a priori based on clinical relevance and previous literature rather than by automated statistical screening. In the NHANES cohort, covariates included demographics (age, sex, race, education, marital status, poverty-to-income ratio [PIR]), lifestyle factors (smoking, alcohol use), anthropometrics (BMI), and comorbidities (hypertension, diabetes, CKD, CVD, MetS). For the hospital cohort, the primary adjusted model included age, sex, and BMI. Additional available clinical and laboratory variables were summarized descriptively in Table 1, but variables such as hypertension, diabetes, CKD, CVD, and MetS were not included in the primary validation model because they are components or close proxies of advanced CKM staging. Variable definitions in the hospital cohort were harmonized with NHANES protocols to maximize comparability.

**TABLE 1:** Baseline characteristics by CALLY index categories defined using NHANES quartile cut points in NHANES and an external hospital cohort.

Characteristics	CALLY index				*P*-value
	Q1 [0.02, 1.50]	Q2 (1.50, 3.64]	Q3 (3.64, 9.13]	Q4 (9.13, 173.90]	
NHANES cohort					
Age (years)	49.64 (16.89)	48.86 (16.50)	47.21 (16.29)	41.60 (15.72)	<0.001***
Sex, (%)					<0.001***
Male	1,065 (36.02)	1,334 (45.15)	1,636 (54.89)	1,714 (57.05)	
Female	1,859 (63.98)	1,588 (54.85)	1,285 (45.11)	1,209 (42.95)	
Race/Ethnicity, (%)					<0.001***
Mexican American	603 (7.53)	591 (7.21)	627 (8.24)	530 (7.07)	
Other Hispanic	152 (4.19)	164 (5.03)	215 (5.17)	184 (4.33)	
Non-Hispanic white	1,463 (71.38)	1,542 (73.98)	1,515 (73.25)	1,531 (72.46)	
Non-Hispanic black	624 (12.83)	547 (10.57)	452 (8.36)	514 (9.23)	
Other races	82 (4.07)	78 (3.21)	112 (4.99)	164 (6.92)	
Education level, (%)					<0.001***
<High school	966 (22.36)	862 (18.73)	858 (18.91)	742 (15.95)	
High school	686 (26.21)	712 (26.80)	730 (26.32)	630 (22.30)	
> High school	1,272 (51.43)	1,348 (54.47)	1,333 (54.77)	1,551 (61.75)	
Drinking alcohol, (%)					<0.001***
Yes	1,819 (66.54)	2,019 (73.78)	2,138 (77.05)	2,222 (78.58)	
No	1,105 (33.46)	903 (26.22)	783 (22.95)	701 (21.42)	
Smoking, (%)					0.011*
Yes	1,440 (51.94)	1,451 (50.45)	1,439 (49.12)	1,352 (46.52)	
No	1,484 (48.06)	1,471 (49.55)	1,482 (50.88)	1,571 (53.48)	
Marital status, (%)					<0.001***
All others	1,139 (36.42)	1,045 (31.19)	1,011 (31.71)	1,130 (35.81)	
Married/living with a partner	1,785 (63.58)	1,877 (68.81)	1,910 (68.29)	1,793 (64.19)	
Hypertension, (%)					<0.001***
Yes	1,227 (40.48)	1,126 (34.04)	959 (29.35)	687 (19.79)	
No	1,683 (59.52)	1,784 (65.96)	1,940 (70.65)	2,217 (80.21)	
Kidney failure, (%)					<0.001***
Yes	134 (3.79)	88 (2.20)	67 (1.58)	39 (1.09)	
No	2,783 (96.21)	2,828 (97.80)	2,851 (98.42)	2,878 (98.91)	
Diabetes, (%)					<0.001***
Yes	405 (11.65)	334 (8.17)	304 (6.76)	195 (4.62)	
No	2,462 (88.35)	2,541 (91.83)	2,566 (93.24)	2,692 (95.38)	
CVD, (%)					<0.001***
Yes	424 (12.25)	350 (9.52)	330 (8.18)	212 (5.29)	
No	2,500 (87.75)	2,572 (90.48)	2,591 (91.82)	2,711 (94.71)	
MetS, (%)					<0.001***
Yes	1,352 (49.06)	1,210 (40.03)	973 (29.46)	528 (15.71)	
No	1,266 (50.94)	1,614 (59.97)	1,900 (70.54)	2,369 (84.29)	
Advanced CKM syndrome, (%)					<0.001***
Yes	734 (19.25)	651 (15.18)	553 (12.62)	354 (7.62)	
No	2,190 (80.75)	2,271 (84.82)	2,368 (87.38)	2,569 (92.38)	
PIR, (%)					<0.001***
≤ 1	651 (15.91)	515 (11.52)	489 (11.66)	508 (11.84)	
1–3	1,278 (39.28)	1,254 (37.17)	1,266 (36.89)	1,154 (33.16)	
> 3	995 (44.81)	1,153 (51.30)	1,166 (51.45)	1,261 (55.00)	
BMI, (%)					<0.001***
<25	482 (16.66)	618 (21.05)	835 (32.12)	1,546 (56.02)	
≥ 25	2,361 (83.34)	2,266 (78.95)	2,055 (67.88)	1,365 (43.98)	
TC, mean (SD), mg/dL	199.00 (41.65)	203.25 (42.39)	200.77 (41.42)	192.27 (37.76)	<0.001***
BUN, mean (SD), mg/dL	13.37 (6.66)	13.34 (5.45)	13.34 (4.96)	12.90 (4.64)	0.012*
Hb, mean (SD), g/dL	13.99 (1.49)	14.51 (1.47)	14.69 (1.45)	14.74 (1.44)	<0.001***
Hospital cohort					
Age (years)	63.64 (14.53)	56.22 (14.04)	55.02 (13.90)	54.08 (14.75)	<0.001***
Sex, (%)					<0.001***
Male	432 (67.39)	82 (58.57)	65 (46.43)	70 (51.85)	
Female	209 (32.61)	58 (41.43)	75 (53.57)	65 (48.15)	
BMI, (%)					0.329
<23	255 (52.80)	56 (44.09)	65 (49.24)	69 (53.08)	
≥ 23	228 (47.20)	71 (55.91)	67 (50.76)	61 (46.92)	
Advanced CKM syndrome, (%)					<0.001***
Yes	421 (65.68)	68 (48.57)	58 (41.43)	47 (34.81)	
No	220 (34.32)	72 (51.43)	82 (58.57)	88 (65.19)	
Hypertension, (%)					<0.001***
Yes	418 (65.21)	78 (55.71)	72 (51.43)	69 (51.11)	
No	223 (34.79)	62 (44.29)	68 (48.57)	66 (48.89)	
Diabetes, (%)					0.729
Yes	124 (19.34)	27 (19.29)	24 (17.14)	21 (15.56)	
No	517 (80.66)	113 (80.71)	116 (82.86)	114 (84.44)	
CVD, (%)					0.885
Yes	174 (27.15)	37 (26.43)	40 (28.57)	33 (24.44)	
No	467 (72.85)	103 (73.57)	100 (71.43)	102 (75.56)	
MetS, (%)					0.382
Yes	175 (27.30)	44 (31.43)	40 (28.57)	30 (22.22)	
No	466 (72.70)	96 (68.57)	100 (71.43)	105 (77.78)	
CKD, (%)					<0.001***
Yes	312 (48.67)	48 (34.29)	34 (24.29)	35 (25.93)	
No	329 (51.33)	92 (65.71)	106 (75.71)	100 (74.07)	
TC, mean (SD), mg/dL	163.27 (65.71)	186.90 (62.69)	178.85 (56.82)	179.46 (59.33)	<0.001***
BUN, mean (SD), mg/dL	12.38 (9.87)	9.44 (9.11)	7.45 (7.20)	6.56 (5.49)	<0.001***
Hb, mean (SD), g/dL	10.31 (2.75)	11.92 (2.66)	11.93 (2.49)	12.45 (2.41)	<0.001***

BMI, body mass index; BUN, blood urea nitrogen; CKD, chronic kidney disease; CVD, cardiovascular disease; Hb, hemoglobin; MetS, metabolic syndrome; PIR, poverty income ratio; Q, quartile; TC, total cholesterol. BMI thresholds were ≥ 25 kg/m^2^ for non-Asian populations and ≥ 23 kg/m^2^ for Asian populations (AHA). NHANES: unweighted n and survey-weighted %. Hospital cohort categorical variables are presented as unweighted n (%), and continuous variables are presented as mean (SD).

*P < 0.05,

***P < 0.001. For variables with missing data, percentages were calculated using participants with non-missing values for that variable.

### 2.4 Statistical analysis

Statistical analyses were performed using R (version 4.4.0). NHANES analyses incorporated sample weights (recalculated for combined cycles), stratification, and clustering to ensure representativeness. Participants were stratified into CALLY index quartiles (Q1–Q4), with NHANES-derived cut-points applied to the external hospital cohort for comparability. Baseline characteristics were presented as mean (SD) and frequency (weighted percentages for NHANES; unweighted for hospital). Weighted logistic regression and Cox proportional hazards models were employed to evaluate associations with advanced CKM (Stages 3–4) and all-cause mortality, respectively. Collinearity among covariates was additionally assessed, and no evidence of problematic multicollinearity was identified. Because Schoenfeld-residual-based diagnostics are not directly implemented for svycoxph, the proportional hazards assumption for the mortality analysis was additionally evaluated in a companion weighted Cox model with the same covariate structure using cox.zph. Potential non-linear relationships were explored using restricted cubic splines (RCS) with three knots. Three knots were chosen to allow flexible modeling of potential nonlinearity while maintaining parsimony and reducing the risk of overfitting, particularly in the smaller external validation cohort. Knot placement was data-driven within the rms framework based on the distribution of the standardized CALLY variable. Model performance was assessed via AUC, NRI, IDI, and decision curve analysis (DCA), with external validation conducted using adjusted logistic regression. All tests were two-sided, and a *P*-value < 0.05 was considered statistically significant.

## 3 Results

### 3.1 Baseline characteristics of the study participants

A total of 11,690 participants from NHANES were included and stratified by CALLY index quartiles (Table 1). The weighted prevalence of advanced CKM syndrome was 19.61%, showing significant differences across quartiles (*P* < 0.001). Compared to Q1, participants in Q4 were younger, had a higher proportion of males, higher education levels and PIR, and a higher prevalence of alcohol consumption (all *P* < 0.001). Q4 had the lowest prevalence of metabolic syndrome (MetS), but lower prevalences of hypertension, diabetes, kidney failure, and cardiovascular disease (CVD) (all *P* < 0.001). Regarding biochemical indices, Q4 exhibited higher hemoglobin (Hb) levels and lower total cholesterol (TC) levels (all *P* < 0.001).

In the hospital validation cohort, application of the NHANES-derived CALLY cut-points resulted in an unequal distribution across CALLY categories. The prevalence of advanced CKM decreased from 65.68% in Q1 to 34.81% in Q4 (*P* < 0.001). Higher CALLY categories were also characterized by younger age, lower prevalence of hypertension and CKD, lower BUN levels, and higher Hb levels. BMI category, diabetes, CVD, and MetS did not differ significantly across CALLY categories.

### 3.2 NHANES cohort: association between CALLY index and advanced CKM syndrome

In the primary NHANES cohort, survey-weighted logistic regression analyses demonstrated a consistent inverse association between the CALLY index and the risk of advanced CKM syndrome (Table 2). Multivariable adjustment in Model 3 yielded a significant odds ratio of 0.84 (95% CI: 0.75–0.94, *P* = 0.004) for each 1-SD rise in the CALLY index. In the categorical assessment, we observed a stepwise decline in risk from the lowest to the highest quartile (Q2: OR = 0.80; Q3: OR = 0.74; Q4: OR = 0.70 vs. Q1), with the dose-response relationship supported by trend analysis (P for trend = 0.005).

**TABLE 2:** Multivariable logistic regression analysis of the association between the CALLY index and advanced CKM syndrome in the NHANES discovery cohort and the hospital validation cohort.

CALLY Index	Model 1		Model 2		Model 3	
	OR (95% CI)	*P*−value	OR (95% CI)	*P*−value	OR (95% CI)	*P*−value
NHANES cohort a						
Continuous	0.58 (0.51, 0.66)	<0.001***	0.79 (0.70, 0.89)	<0.001***	0.84 (0.75, 0.94)	0.004 **
Quartiles						
Q1	Ref		Ref		Ref	
Q2	0.76 (0.64, 0.90)	0.002**	0.75 (0.60, 0.94)	0.012*	0.80 (0.64, 1.00)	0.050*
Q3	0.62 (0.53, 0.73)	<0.001***	0.66 (0.54, 0.81)	<0.001***	0.74 (0.60, 0.91)	0.004**
Q4	0.36 (0.29, 0.43)	<0.001***	0.59 (0.46, 0.75)	<0.001***	0.70 (0.54, 0.90)	0.006**
*P* for trend		<0.001***		<0.001***		0.005**
Hospital cohort b						
Continuous	0.86 (0.73, 1.03)	0.093	0.91 (0.79, 1.04)	0.170	0.91 (0.79, 1.04)	0.164
Quartiles						
Q1	Ref		Ref		Ref	
Q2	0.53 (0.36, 0.79)	0.002**	0.62 (0.41, 0.94)	0.024*	0.63 (0.42, 0.95)	0.026*
Q3	0.41 (0.28, 0.61)	<0.001***	0.53 (0.36, 0.79)	0.002**	0.53 (0.36, 0.80)	0.002**
Q4	0.33 (0.22, 0.49)	<0.001***	0.41 (0.28, 0.62)	<0.001***	0.41 (0.28, 0.62)	<0.001***
*P* for trend		<0.001***		<0.001***		<0.001***

Data are presented as odds ratios (OR) with 95% confidence intervals (CIs). Model 1: Unadjusted.

^a^NHANES Cohort Adjustments: Model 2 is adjusted for age, gender, ethnicity, marital status, educational attainment, and poverty income ratio (PIR). Model 3 is further adjusted for smoking status, drinking status, and body mass index (BMI).

^b^Hospital Cohort Adjustments: Model 2 is adjusted for age and gender. Model 3 is further adjusted for BMI. Continuous analyses used the winsorized and standardized (z-score) CALLY index. Effect sizes for continuous analyses are per 1-SD increment.

***P < 0.001,

**P < 0.01,

*P < 0.05. In the hospital cohort, all three logistic regression models were fitted using the same complete-case sample with available CALLY index, advanced CKM status, age, sex, and BMI (*n* = 872).

In the hospital complete-case analysis, the continuous CALLY index was not significantly associated with advanced CKM after full adjustment (OR = 0.91, 95% CI: 0.79–1.04, *P* = 0.164). However, when categorized using NHANES-derived cut-points, higher CALLY categories were associated with lower odds of advanced CKM. Compared with Q1, participants in Q2, Q3, and Q4 had lower odds of advanced CKM in the fully adjusted model (Q2: OR = 0.63, 95% CI: 0.42–0.95, *P* = 0.026; Q3: OR = 0.53, 95% CI: 0.36–0.80, *P* = 0.002; Q4: OR = 0.41, 95% CI: 0.28–0.62, *P* < 0.001), with a significant ordinal trend across categories (P for trend <0.001).

**FIGURE 2:**
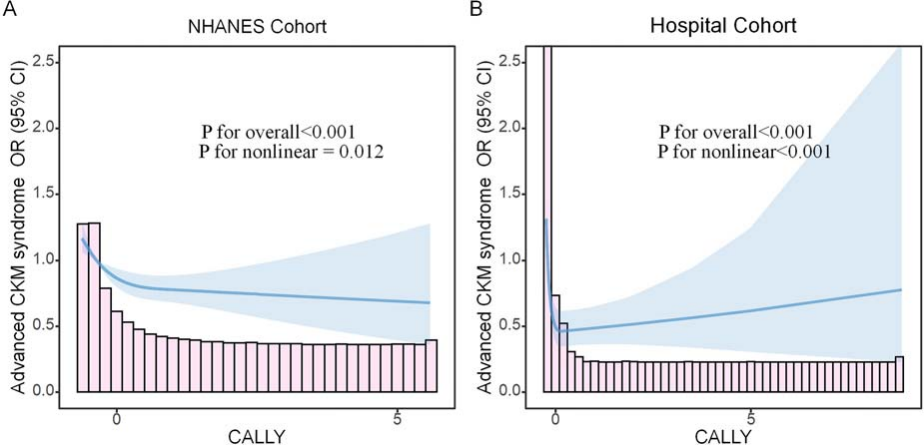
The nonlinear associations between the CALLY index and advanced CKM syndrome. The solid blue line represents the smooth curve fit between variables. Blue bands represent the 95% confidence interval from the fit. **(A)** NHANES cohort. **(B)** Hospital cohort.

Notably, restricted cubic spline (RCS) analyses revealed comparable non-linear dose-response patterns in the two cohorts (Figure 2). In NHANES (Figure 2A), the risk decreased steeply with increasing CALLY levels at lower ranges before plateauing (P for overall <0.001; P for non-linearity = 0.012). This characteristic “L-shaped” curve was mirrored in the hospital cohort (Figure 2B), which also demonstrated significant non-linearity (P for overall <0.001; P for non-linearity <0.001).

### 3.3 Incremental predictive value and clinical utility

**FIGURE 3:**
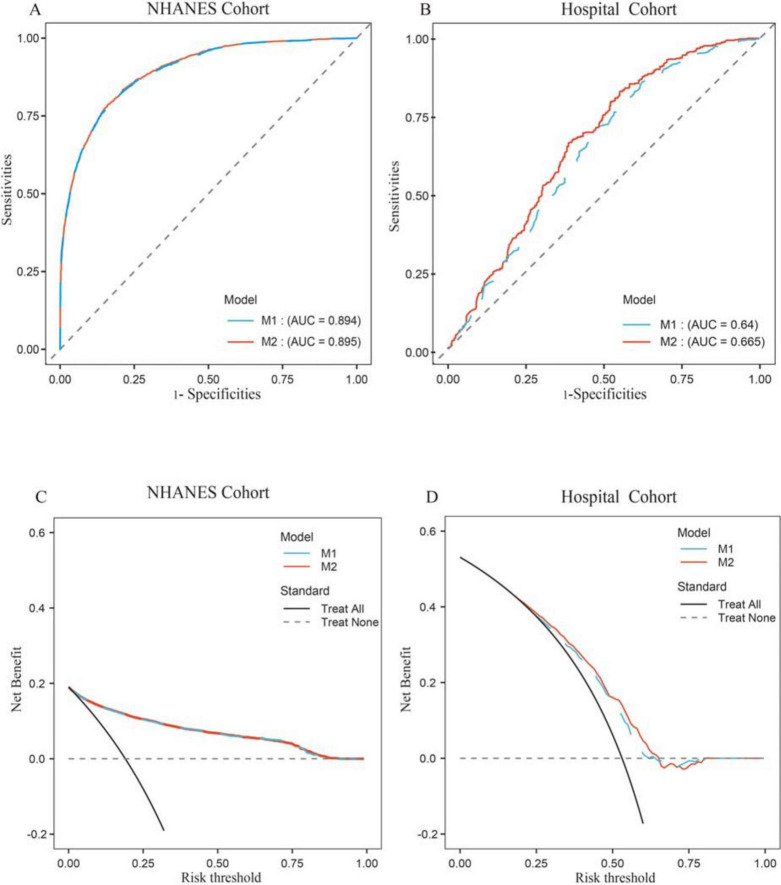
Predictive performance and clinical utility. **(A,B)** ROC curves comparing the baseline model (M1, blue line) and the CALLY-integrated model (M2, red line) in the NHANES **(A)** and hospital **(B)** cohorts. **(C,D)** Decision curve analyses (DCA) illustrating the net benefit of M1 versus M2 in the NHANES **(C)** and hospital **(D)** cohorts. Black and grey dashed lines represent “treat-all” and “treat-none” strategies, respectively. AUC, area under the curve; NRI, net reclassification improvement; IDI, integrated discrimination improvement; M1, Baseline Model (adjusted for age, sex, and BMI); M2, Baseline + CALLY index Model.

We evaluated the incremental predictive value of the CALLY index across both cohorts (Figure 3). In the NHANES discovery cohort (Figure 3A), adding the CALLY index to the baseline model (M1) yielded a statistically significant, albeit marginal, increase in AUC (0.895 vs. 0.894, *P* = 0.001 by DeLong test). Reclassification analyses further showed improvement in risk stratification, with a Net Reclassification Improvement (NRI) of 0.1968 (*P* < 0.001) and an Integrated Discrimination Improvement (IDI) of 0.0031 (*P* < 0.001).

In the external hospital validation cohort (Figure 3B), the CALLY-integrated model (M2) demonstrated modest discrimination improvement over baseline (AUC: 0.665 vs. 0.640, *P* = 0.018 by DeLong test). This was supported by marked reclassification gains, with an NRI of 0.4134 (*P* < 0.001) and an IDI of 0.0247 (*P* < 0.001). Decision Curve Analysis (Figures 3C,D) suggested greater model-based net benefit for the CALLY-integrated model, particularly in the hospital cohort, where it provided higher net benefits across pivotal threshold probabilities (20–60%).

### 3.4 Association between CALLY index and all-cause mortality risk

**FIGURE 4:**
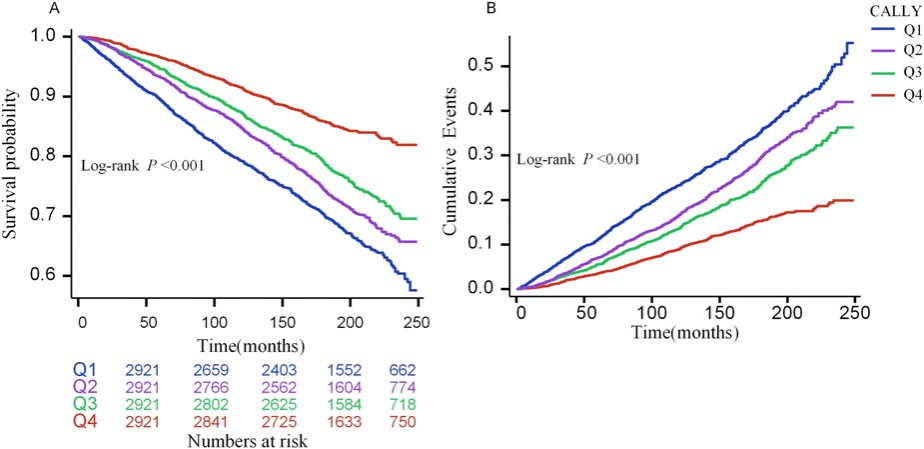
Kaplan-Meier analysis of all-cause mortality in the NHANES cohort. **(A)** Survival probability curves and **(B)** cumulative event curves stratified by CALLY index quartiles. The blue, purple, green, and red lines represent Q1, Q2, Q3, and Q4, respectively.

Survival analysis was restricted to the NHANES cohort. Over a median follow-up of 157.0 months (total 152,322.7 person-years), 2,583 all-cause deaths were recorded among 11,684 participants, corresponding to an incidence rate of 16.96 per 1,000 person-years. Kaplan-Meier survival analysis demonstrated clear risk stratification (Figure 4), with survival probability progressively increasing from the lowest (Q1) to the highest (Q4) CALLY quartile (Log-rank *P* < 0.001). In the fully adjusted survey-weighted Cox proportional hazards model (Table 3), higher CALLY index levels were independently associated with a significantly reduced mortality risk compared to Q1 (Q2: HR = 0.77, 95% CI: 0.68–0.88; Q3: HR = 0.65, 95% CI: 0.56–0.74; Q4: HR = 0.64, 95% CI: 0.55–0.76; P for trend <0.001). Additional diagnostic analyses suggested time-varying effects for some terms, including the CALLY quartile variable, and the global test was also statistically significant. Therefore, the mortality hazard ratios should be interpreted as average relative risk estimates over follow-up rather than strictly time-invariant effects. These findings were generally consistent across unadjusted and demographic-adjusted models, supporting an inverse association between a higher CALLY index and all-cause mortality.

**TABLE 3:** Weighted Cox proportional hazards regression analysis of the association between the CALLY index and all-cause mortality among adults in the NHANES cohort.

CALLY index	Model 1		Model 2		Model 3	
	HR (95% CI)	*P*−value	HR (95% CI)	*P*−value	HR (95% CI)	*P*−value
Q1	Ref		Ref		Ref	
Q2	0.70 (0.62–0.81)	<0.001***	0.75 (0.66–0.85)	<0.001***	0.77 (0.68–0.88)	<0.001***
Q3	0.53 (0.47–0.60)	<0.001***	0.64 (0.56–0.73)	<0.001***	0.65 (0.56–0.74)	<0.001***
Q4	0.33 (0.28–0.38)	<0.001***	0.62 (0.54–0.72)	<0.001***	0.64 (0.55–0.76)	<0.001***
*P* for trend		<0.001***		<0.001***		<0.001***

Data are presented as weighted hazard ratios (HRs) with 95% confidence intervals (CIs). Model 1: Unadjusted. Model 2: Adjusted for age, gender, ethnicity, marital status, educational attainment, and poverty income ratio (PIR). Model 3: Further adjusted for smoking status, drinking status, and body mass index (BMI) based on Model 2.

***P < 0.001.

### 3.5 Subgroup analyses

In the NHANES cohort, the inverse association between CALLY index and advanced CKM syndrome was consistent across subgroups defined by sex, race, marital status, education, smoking status, alcohol consumption, hypertension, diabetes, and BMI, with no significant interactions (all P for interaction > 0.05; Supplementary Figure 2A). The association appeared to be attenuated or reversed in the subgroup with established CVD (OR = 1.56; 95% CI: 0.95, 2.54), although the interaction test was not statistically significant (P for interaction = 0.10). In the external hospital cohort, the directions of associations across most subgroups were generally consistent with those in NHANES, and most interaction terms were not significant (P for interaction > 0.05; Supplementary Figure 2B). Stratified analyses revealed that sex significantly altered the association (P for interaction = 0.02). A stronger inverse association trend was observed in women (OR = 0.56, 95% CI: 0.31–1.03), whereas no clear association was observed in men (OR = 0.96, 95% CI: 0.82–1.11). However, because the confidence interval in women crossed 1.0, this sex-specific finding should be interpreted with caution.

## 4 Discussion

Using data from the large-scale NHANES survey and an independent clinical validation cohort, our findings support an independent inverse association between the CALLY index and the prevalence of advanced CKM syndrome. Recent NHANES-based studies have begun to evaluate the CALLY index in CKM-related settings, and our study extends this emerging literature by jointly examining advanced CKM and all-cause mortality within one framework while externally validating the advanced CKM association in an independent real-world hospital cohort. Our analysis revealed a consistent “L-shaped” nonlinear dose-response pattern in both populations, characterized by a steep reduction in risk at lower index levels that subsequently plateaus. Expanding our scope to a longitudinal setting, we observed that the CALLY index was independently associated with all-cause mortality in the NHANES cohort. Beyond standard associations, our use of reclassification metrics (NRI and IDI) and DCA demonstrates that adding the CALLY index to standard risk models improves risk stratification accuracy and enhances clinical net benefit.

Recent NHANES-based studies have separately linked the CALLY index to advanced CKM syndrome, with emphasis on age-related heterogeneity and threshold effects, and to mortality among individuals with CKM stages 0–3 (20, 21). Our findings are complementary to these reports, but extend this literature by focusing on late-stage CKM risk stratification, integrating disease severity and prognosis within one framework, evaluating potential clinical usefulness through AUC, NRI/IDI, and DCA, and testing the applicability of the advanced CKM association in an independent Chinese hospital-based cohort with a different ethnic composition, case mix, and data structure.

Our results corroborate and extend earlier research, which has primarily focused on isolated cardiovascular or metabolic outcomes. For instance, Ji et al. (23) linked the CALLY index to adverse events in patients with STEMI, while Wu et al. (24) identified an association with peripheral artery disease risk. Similarly, Luo et al. (25) validated its prognostic value for mortality in the elderly. While these studies highlighted the index’s value in specific conditions, our work demonstrates its relevance to the broader CKM syndrome, a complex construct involving metabolic, renal, and cardiovascular interactions. Consistent with Zheng et al. and Huang et al., who emphasized the importance of the inflammation–immune axis in CKM prognosis (26, 27), our subgroup analyses showed consistent trends across diverse groups based on sex, race, and metabolic status. A possible sex-related difference was observed in the external hospital cohort, with a stronger inverse association trend in women than in men. However, this finding should be interpreted cautiously because the confidence interval in women crossed the null value and the subgroup sample size was limited. Potential explanations may include sex-related differences in inflammatory regulation, immune response, body composition, and nutritional reserve. Prior studies have suggested that hormonal and immune-inflammatory pathways may differ by sex, which could influence the composite behavior of CRP, albumin, and lymphocyte count. Nevertheless, residual confounding and chance cannot be excluded, and this observation should be regarded as hypothesis-generating rather than conclusive. However, the trend toward a reversed association in patients with established CVD suggests that once end-stage organ damage occurs, the clinical interpretation of the CALLY index may need to be adjusted (28). This altered association in established CVD patients may be attributed to the widespread use of secondary prevention medications (e.g., statins, antiplatelets) which modulate inflammatory pathways, or the “obesity paradox” phenomenon often observed in end-stage cardiac diseases.

The association we observed is biologically plausible, as the CALLY index effectively captures the “inflammation-nutrition-immunity axis,” reflecting the body’s systemic defense capacity (15, 16). The progression of CKM syndrome is driven by the interplay of chronic inflammation, oxidative stress, and metabolic dysregulation (6). Mechanistically, a low CALLY index, signaling high inflammatory burden and nutritional depletion, may exacerbate vascular endothelial injury, glomerulosclerosis, and myocardial remodeling, thereby accelerating the transition to advanced disease stages (29–31). In contrast, a high CALLY index suggests a stronger multi-dimensional biological defense: elevated albumin serves as a critical thiol donor, providing an antioxidant barrier against lipid peroxidation (32, 33); controlled CRP levels reflect suppressed systemic inflammation, inhibiting vascular remodeling (32); and adequate lymphocyte counts maintain immune surveillance and tissue repair capacity (34). This systemic advantage of “anti-inflammation, high nutrition, and immune homeostasis” likely buffers metabolic stress (35, 36), impeding the pathological trajectory toward end-stage organ damage (37, 38).

The robustness of the findings and the incremental predictive value of the CALLY index are important strengths of this study. While the inverse trend held true across both cohorts, we noted distributional differences in the hospital cohort, where statistical significance was concentrated in the higher quartiles with wider confidence intervals. These variations likely stem from the smaller sample size, greater disease severity among hospitalized patients, and differences in assay platforms (39, 40). Nevertheless, the persistence of the nonlinear relationship in both cohorts suggests that the inflammatory-nutritional balance is a fundamental mechanism in CKM progression, regardless of the population setting (41). Crucially, our study offers new evidence regarding the incremental predictive value of the CALLY index. Although the improvement in AUC was marginal, this does not necessarily imply limited clinical relevance. AUC reflects overall discrimination and may be relatively insensitive to the addition of a single biomarker when the baseline model already performs well, whereas NRI and IDI are more informative for assessing improvement in individual-level risk allocation (42). This suggests that the CALLY index captures residual risk information related to immune-nutritional status that may not be fully reflected by traditional risk factors, thereby improving risk reclassification for a proportion of participants. Furthermore, DCA showed that the CALLY-integrated model provided greater net benefit than the standard model, particularly in the clinical validation cohort, supporting its potential clinical usefulness (43).

Regarding long-term outcomes, survival analysis showed that a higher CALLY index was independently associated with lower all-cause mortality in the NHANES cohort, extending its prognostic relevance beyond oncology (44, 45). The “L-shaped” dose-response curve has important clinical implications. It identifies a potential “window of opportunity” for patients in the lower index range (the steep portion of the curve). For these high-risk or early-stage CKM patients, targeted interventions, such as anti-inflammatory strategies, dietary optimization, and protein supplementation, may yield the greatest marginal benefit (46–49). Such timely interventions could potentially slow disease progression to advanced stages (50, 51) and improve survival trajectories (13, 52, 53), providing a theoretical basis for establishing CALLY-based intervention thresholds.

## 5 Limitations

Our study has several limitations. First, the cross-sectional nature of the NHANES data prevents causal inference regarding CKM progression. Second, CKM staging relied on surrogate measures like the PREVENT equations (54), which may not fully capture the complex pathology of cardiorenal metabolic impairments. In addition, because several CKM stage components in the hospital cohort were derived from EHR-based proxy measures rather than direct research-grade variables, some degree of misclassification cannot be excluded. Third, the complete-case approach used in the NHANES analysis may have introduced selection bias by preferentially retaining participants with more complete laboratory and questionnaire data. However, the use of survey weights and the consistency of findings in the external validation cohort partly support the robustness of the results. Fourth, the hospital cohort was limited by sample size and available covariates. Although additional clinical and laboratory variables were summarized in Table 1, socioeconomic and lifestyle factors, such as smoking, alcohol use, education, and income, were not routinely available in the EHR. In addition, some variables, including BMI, had missing values; therefore, the hospital validation models were based on complete-case analyses, which may have introduced residual confounding or selection bias. Moreover, diagnostic testing suggested that the proportional hazards assumption was not fully supported for all terms in the mortality model, indicating possible time-varying effects over follow-up. Therefore, the reported hazard ratios should be interpreted as average relative risk estimates over the study period. Future longitudinal studies are needed to validate causal pathways and define clinically applicable thresholds, facilitating the integration of the CALLY index into routine personalized care for cardiorenal metabolic syndrome.

## 6 Conclusion

In the representative NHANES cohort, the CALLY index was independently and inversely associated with advanced CKM and all-cause mortality. In the external hospital cohort, higher CALLY categories were also associated with lower odds of advanced CKM, supporting the cross-population transportability of the advanced CKM association. Restricted cubic spline analysis revealed a characteristic “L-shaped” relationship, suggesting that improvements in the low CALLY index range may yield the greatest marginal benefit. As a simple, low-cost, and readily accessible composite biomarker, CALLY index can be utilized for risk stratification across the CKM continuum and for early identification of high-risk populations. Given the observational nature of this study, prospective and interventional research is still required to calibrate CALLY index thresholds and evaluate the real-world clinical benefits of improving its constituent components.

## Data Availability

NHANES data are publicly available. Hospital cohort data are not publicly available due to privacy and ethical restrictions, but may be available from the corresponding authors upon reasonable request and institutional approval.
