# Supplementary material for: The CALLY index for risk stratification of advanced cardiovascular-kidney-metabolic syndrome and mortality: a dual-cohort study from NHANES and a real-world clinical setting
Source: Front Med (Lausanne). 2026 Jun 26;13:1811091. doi: 10.3389/fmed.2026.1811091 (PMC13352524; doi:10.3389/fmed.2026.1811091)
Supplement: Supplementary file 1 [file Table_1.docx]

Supplementary Material

## Supplementary Figures

**
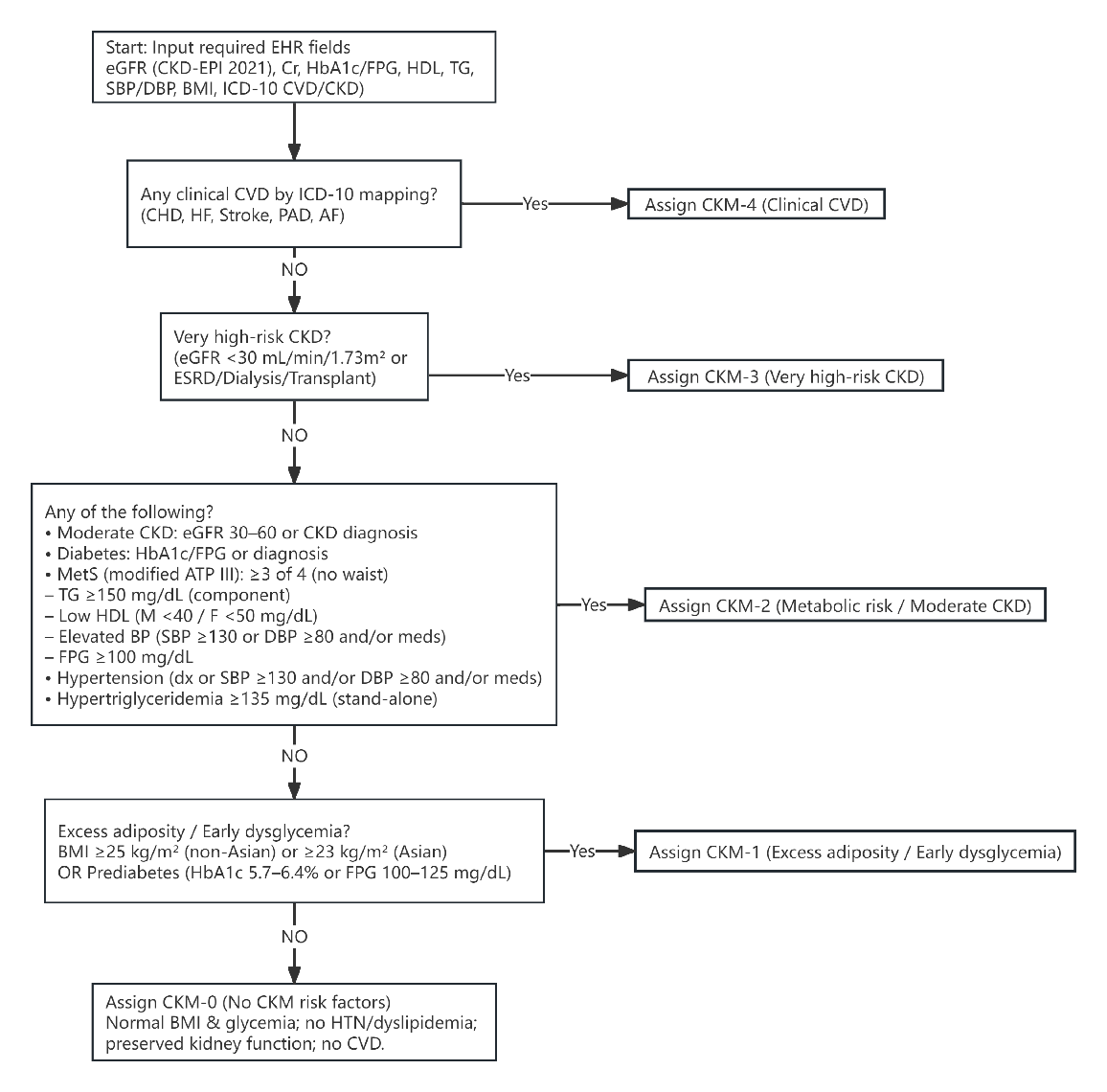
**

**Supplementary Figure 1.** **Flowchart of the hierarchical CKM staging algorithm and data processing workflow in the external hospital cohort.**

The CKM staging was performed using a top-down hierarchical algorithm (Stage 4→Stage 0); participants were assigned to the highest qualifying stage, and lower stages were not evaluated once a higher stage criterion was met. Diagnoses for cardiovascular disease (CVD) and chronic kidney disease (CKD) were identified using ICD-10 concept sets derived from electronic health records. An Asian-specific body mass index (BMI) cutoff (≥23 kg/m²) was consistently applied. Data processing involved unit harmonization and person-level de-duplication based on the index
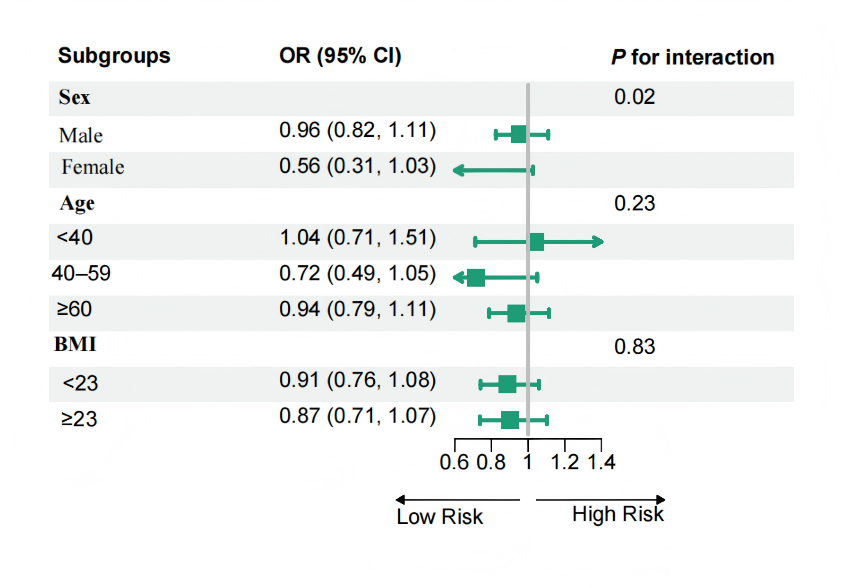

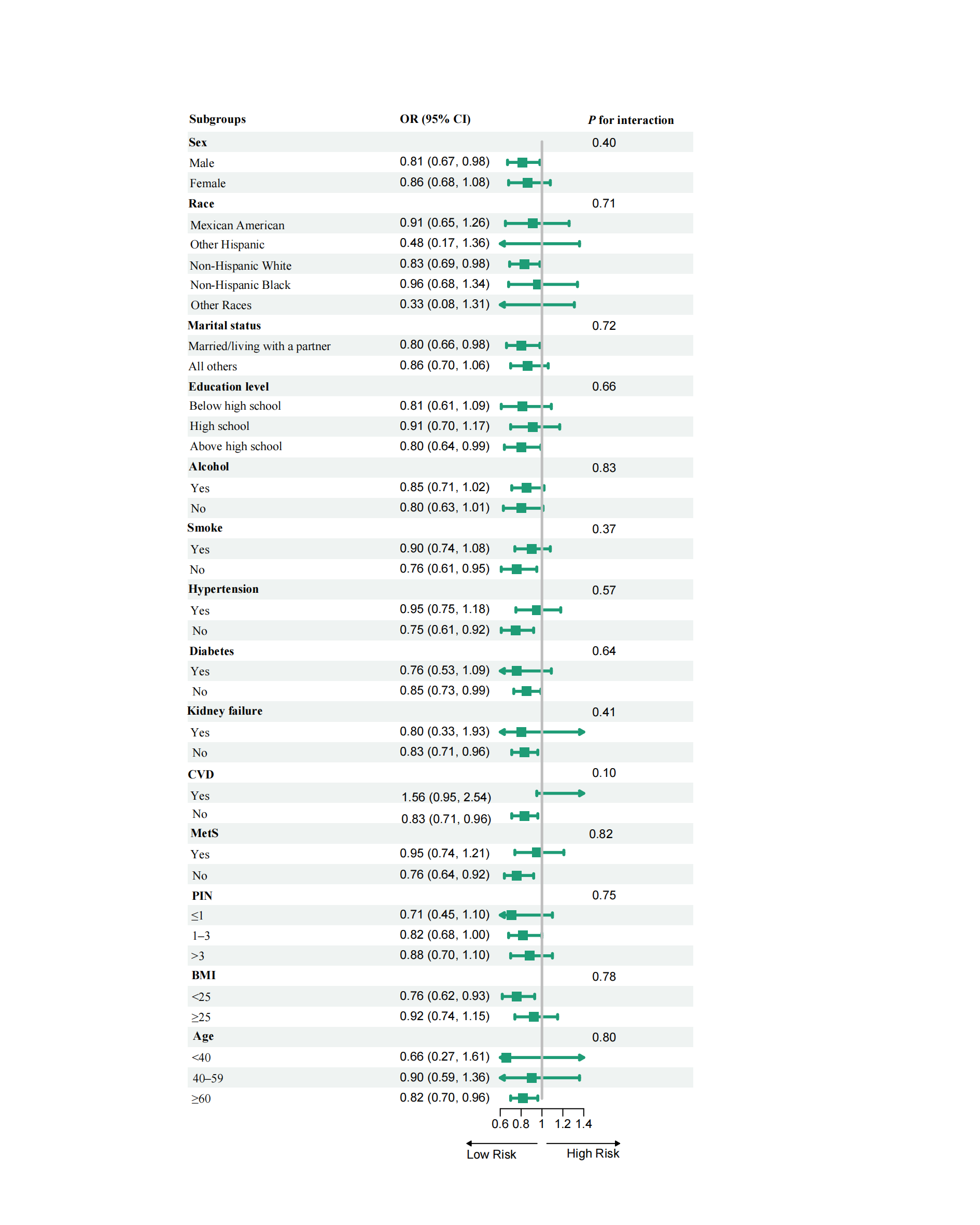
admission date.

1. (B)

**Figure S2. Subgroup analyses of the association between the CALLY index and advanced CKM syndrome.**

(A) Forest plot of subgroup analysis in the NHANES cohort. Stratification factors included sex, race, marital status, education level, alcohol consumption, smoking status, hypertension, diabetes, kidney failure, cardiovascular disease (CVD), metabolic syndrome (MetS), poverty income ratio (PIR), body mass index (BMI), and age.

(B) Forest plot of subgroup analysis in the external hospital cohort, stratified by sex, age, and BMI. The data are presented as odds ratios (ORs) and 95% confidence intervals (CIs). *P* for interaction indicates the statistical significance of the interaction between the CALLY index and each subgroup variable.

# Supplementary Methods: CKM Staging Algorithm and Data Processing

## Scope and index date.

CKM staging used a top-down hierarchical algorithm (Stage 4 → Stage 0), as shown in Figure S1. Baseline data were taken from the first hospitalization (index admission); when multiple laboratory results were available within that admission, the value closest to the admission time was used. Person-level de-duplication was applied.

## Data inputs and harmonization.

Required inputs were eGFR (CKD-EPI 2021 from serum creatinine), fasting plasma glucose (FPG), HDL-C, triglycerides (TG), systolic/diastolic blood pressure (SBP/DBP), BMI, and clinical diagnoses. Units were harmonized (e.g., glucose mmol/L → mg/dL), and biologically implausible values were excluded.

## Diagnosis ascertainment.

Coronary heart disease (CHD), heart failure (HF), stroke, peripheral artery disease (PAD), CKD, and hypertension were identified primarily via ICD-10 concept sets in the EHR; fuzzy keyword matching of discharge summaries was used only as a backup.

## Hospital implementation.

**Stage 3.** Very high-risk CKD (eGFR <30 mL/min/1.73 m² or ESRD/dialysis) was used, given the lack of routine subclinical cardiac screening (CAC/biomarkers) in the EHR.

**Metabolic syndrome (MetS).** Defined as ≥3 of 4 components—low HDL-C (men <40 mg/dL; women <50 mg/dL), TG ≥150 mg/dL, elevated BP (SBP ≥130 mmHg and/or DBP ≥80 mmHg or antihypertensive medication use), or FPG ≥100 mg/dL—excluding waist circumference due to unavailability. HbA1c was not used as a MetS component.

**Stage 2 classification.** Assigned when Stage 3/4 criteria were not met but any of the following were present: moderate CKD (eGFR 30–60 mL/min/1.73 m²), diabetes, MetS, hypertension, or isolated hypertriglyceridemia (≥135 mg/dL).

**Glycemic definitions.**
Prediabetes (Stage 1): HbA1c 5.7%–6.4% or FPG 100–125 mg/dL.
Diabetes (Stage 2): clinical diagnosis, HbA1c ≥6.5%, or FPG ≥126 mg/dL; either laboratory criterion was sufficient when records were valid.

## Clinical adjudication (quality control).

No separate statistical sensitivity analyses were performed. To minimize misclassification, two attending clinicians independently reviewed and adjudicated algorithm-assigned stages against the source EHR for all Stage 3–4 cases and for a stratified random sample of Stage 0–2 cases. Discrepancies were resolved by consensus, and the adjudicated stage was retained for analysis.

## Cross-cohort harmonization.

Rule prioritization, ICD-10 mapping, and BMI cutoffs (non-Asian ≥25 kg/m²; Asian ≥23 kg/m²) were aligned with NHANES protocols to ensure comparability.

## Auditability.

The algorithm output included the final stage, the primary rationale, and component flags (e.g., Diabetes_flag, MetS_flag) to enable case-level verification.

# Supplementary Table S1. Proportional hazards diagnostics for the mortality model using Schoenfeld residuals

| **Term** | **Chi-square** | **df** | **P‐value** |
| --- | --- | --- | --- |
| CALLY index quartile | 60620 | 3 | <0.001*** |
| Age (years) | 12779 | 1 | <0.001*** |
| Sex | 44681 | 1 | <0.001*** |
| Race/ethnicity | 17990 | 4 | <0.001*** |
| Marital status | 40750 | 5 | <0.001*** |
| Education level | 9842 | 4 | <0.001*** |
| PIR | 17956 | 1 | <0.001*** |
| Smoking | 22198 | 1 | <0.001*** |
| Drinking alcohol | 7605 | 1 | <0.001*** |
| BMI | 11324 | 1 | <0.001*** |
| Global test | 256009 | 22 | <0.001*** |

**Note:** The proportional hazards assumption was evaluated in a companion weighted Cox model with the same covariate structure as the primary survey-weighted Cox model using Schoenfeld residuals (cox.zph). The global test and the CALLY index quartile both suggested evidence of time-varying effects.

# Supplementary Table S2. Collinearity diagnostics for covariates included in the final mortality model

| **Term** | **GVIF** | **Df** | **GVIF^(1/(2×Df))** |
| --- | --- | --- | --- |
| Sex | 1.1354 | 1 | 1.0656 |
| Race/ethnicity | 1.3961 | 4 | 1.0426 |
| Marital status | 1.2403 | 5 | 1.0218 |
| Education level | 1.6198 | 4 | 1.0621 |
| PIR | 1.4254 | 1 | 1.1939 |
| Smoking | 1.1584 | 1 | 1.0763 |
| Drinking alcohol | 1.1992 | 1 | 1.0951 |
| BMI | 1.0354 | 1 | 1.0175 |

**Note:** Low GVIF values and adjusted GVIF^(1/(2×Df)) values close to 1 indicate no evidence of problematic multicollinearity among covariates in the final mortality model.
